# A Case of Subclavian Artery Thrombosis

**DOI:** 10.7759/cureus.6842

**Published:** 2020-02-01

**Authors:** Syed Adeel Hassan, Ali Akhtar, Noor Ul Falah, Maham Khan, Urooj Zahra

**Affiliations:** 1 Internal Medicine, Dow University of Health Sciences, Karachi, PAK; 2 Internal Medicine, Army Medical College, National University of Medical Sciences, Rawalpindi, PAK; 3 Internal Medicine, King Edward Medical University, Lahore, PAK; 4 Radiology, Armed Forces Institute of Radiology and Imaging, Rawalpindi, PAK; 5 Internal Medicine, Fatima Jinnah Medical University, Lahore, PAK

**Keywords:** chronic smoker, undiagnosed diabetes, unmanaged diabetes, right sublavian artery occlusion, gangrene, amputation, upper extremity thrombosis, symptomatic, blood supply

## Abstract

Subclavian artery thrombosis is a rare cause of upper limb ischemia resulting from occlusion of the upper extremity blood supply. Symptomatic presentation is quite rare and therefore remains underdiagnosed by physicians. Possible catastrophic clinical consequences necessitate prompt rectification of the underlying disease and risk factors. Treatment modalities are often selected depending on the severity of clinical presentation. Herein, we present a case of a 52-year-old man who presented to the outpatient department with a one-month history of pain and blackish discoloration of the right-hand digits, palm, and wrist. His social history also revealed a chronic 30-year history of smoking. At the time of consultation, his past medical history was insignificant for chronic medical disease and hospitalizations. However, inpatient investigations diagnosed him with diabetes. Physical examination revealed a bad odor emanating from the devitalized affected right hand. Neurological examination was significant for the loss of pain sensation in the right hand. Furthermore, the right radial and brachial pulses were also absent (grade 0). Right upper extremity angiography revealed the occlusion of the right subclavian artery and right brachial artery. Above elbow amputation was advised and performed based on angiogram scans and physical examination findings. We report a case of subclavian artery thrombosis in an undiagnosed diabetic with a chronic history of smoking. Our report details the common etiology, clinical presentation, and management options feasible for this clinical entity. Furthermore, it reiterates the importance of counseling patients to attend annual healthcare doctor visits.

## Introduction

Subclavian arteries are a pair of arteries that supply arterial blood to the right and left upper extremities. The left subclavian artery arises from the arch of the aorta, while the right subclavian arises from the bifurcation of the innominate artery. Like other arteries, it can also be victimized by pathology. Subclavian artery thrombosis presents in <1% of the population. Therefore, symptomatic lesions are rare in the population. As a consequence, it remains underdiagnosed by physicians. The subclavian occlusion occurs due to vessel wall intimal damage. The common causes of intimal damage include atherosclerosis, external muscular compression, trauma, and repetitive stress. There is a fourfold predilection for thrombosis of the left subclavian artery as compared to its right counterpart [1]. Clinical symptoms are known to occur secondary to diminished blood flow to the upper extremity.

## Case presentation

A 52-year-old man presented to the outpatient department with a one-month history of pain and blackish discoloration of the right-hand digits, palm, and wrist. The pain was continuous, slow, and aching in character. It preceded the blackish discoloration of the hand. Social history revealed a chronic 30-year history of smoking. Furthermore, the patient does not consume alcohol and had no past medical history of hospitalizations or chronic illnesses such as diabetes, hypertension, heart disease, or stroke.

On admission, he was afebrile, with blood pressure 100/70 mmHg, heart rate 103 beats per minute, oxygen saturation 97% in room air, and a respiratory rate of 21/minute. Physical examination was consistent for a bad odor with gangrene of the right-hand digits and palm, which extended till his right wrist. Neurological examination revealed a loss of pain sensation in the affected area. Radial and brachial pulses were notably absent. Laboratory investigations conducted at the hospital diagnosed him with diabetes with elevated HbA1C and abnormal estimated average glucose. Complete blood count was consistent with anemia, leukocytosis, neutrophilia, monocytosis, eosinophilia, and thrombocytosis. Coagulation profile results were also within normal limits. Electrocardiogram did not reveal any abnormalities. Table 1 provides a detailed breakdown of laboratory investigations.

**Table 1:** Patients' diagnostic test results

Investigation	Result
Glycated hemoglobin	6.87%
Estimated average glucose	150 mg/dL
Hemoglobin	11.6 g/dL
Total leukocyte count	15.64 x 10^9/L
Neutrophil count	12.66 x 10^9/L
Monocyte count	1.02 x 10^9/L
Eosinophil count	0.60 x 10^9/L
Platelet count	623,000
Prothrombin time	11.01 seconds
International normalized ratio	1.8
Activated partial thromboplastin time	30.6 seconds

**Figure 1:**
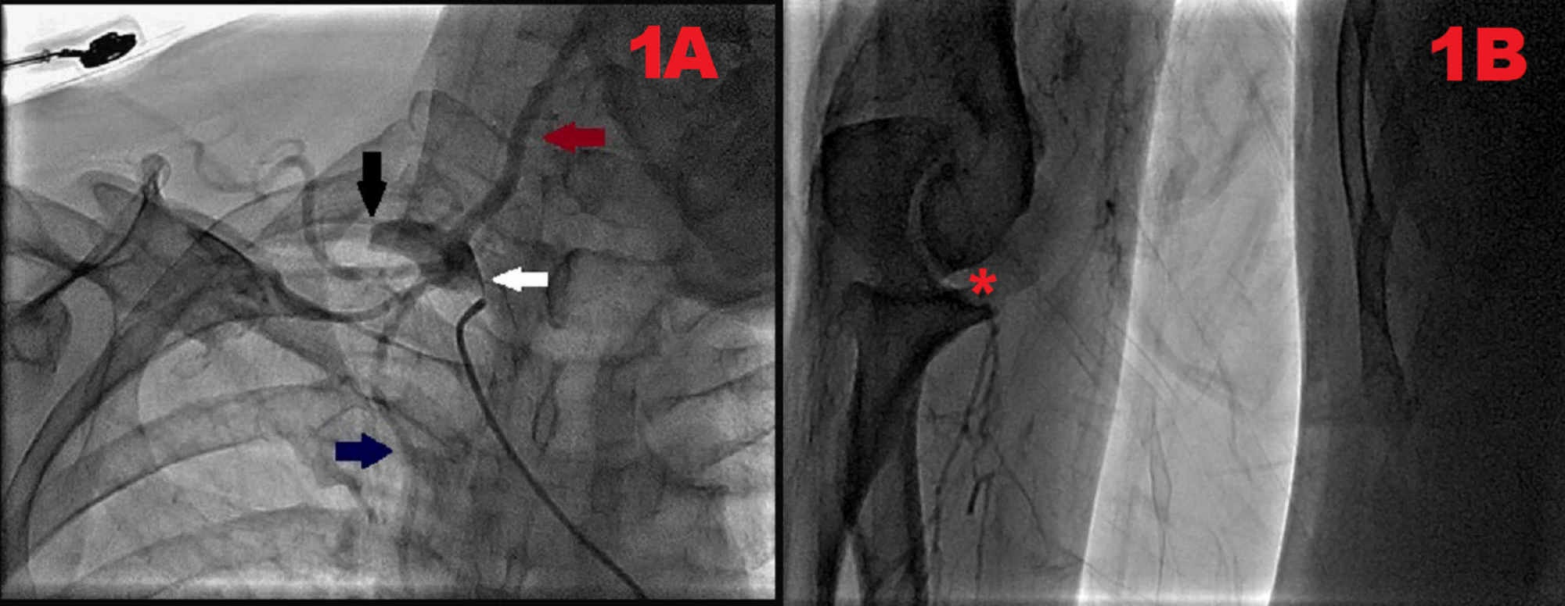
Right upper extremity angiogram depicting occlusion of the right subclavian artery and right brachial artery, respectively. (A) The origin of the right subclavian artery (white arrow) and its complete proximal occlusion (black arrow) can be visualized. Vertebral artery (maroon arrow) and internal thoracic artery (blue arrow) can also be seen arising from the right subclavian artery. (B) Right brachial artery occlusion at the level of the elbow (red asterisk) with poor distal filling can be seen.

Furthermore, transthoracic echocardiography revealed a normal systolic and diastolic function. An ejection fraction of 70% was also noted with no valvular pathology. Right upper extremity angiography was performed, which revealed the occlusion of the right subclavian artery in its course after giving off the vertebral and internal thoracic branches (Figure 1A). Distal filling of the axillary artery via collateral branches was also evident. The right brachial artery was occluded at the level of the elbow, along with poor distal filling (Figure 1B). Hence, the above-elbow amputation of the right upper extremity was advised and performed. The patient was further referred to an endocrinologist for strict diabetes management. Furthermore, he was strongly counseled and scheduled for smoking cessation classes and provided with a stringent follow-up care routine.

## Discussion

Upper extremity ischemia due to thrombosis of the subclavian artery is quite uncommon. Symptomatic lesions tend to occur in <1% of the population [1]. There is a fourfold predilection for thrombosis of the left subclavian artery as compared to its right counterpart [1]. Autopsy series have indicated occlusion of the left subclavian artery in 9% of the population [2]. The main risk factors for the development of thrombosis include obesity, hypertension, trauma, diabetes, and smoking [1,2]. Occlusion occurs as a result of co-existing hypercoagulable states, shear stress, or damage to the vessel intimal lining. The underlying damage promotes platelet aggregation and the release of platelet-derived growth factors [1]. The proliferation of smooth muscle cells follows and results in the formation of an atherosclerotic plaque.

Atherosclerosis is the most common etiology facilitating thrombosis [1]. Atheroma formation tends to occur most commonly in the carotid-subclavian and carotid-vertebral areas [3,4]. Therefore, these anatomical areas are more likely to be involved in the occlusive disease process. Occlusion secondary to atherosclerosis is clinically insidious. It only presents with symptoms when a critical degree of occlusion is achieved. Subclavian thrombosis can also be due to regional anatomical abnormalities, exertional activities, and muscle enlargement. In such instances, damage to the intimal wall occurs as a result of external compression and shear [3]. Other uncommon causes include trauma, radiation exposure, fibro-muscular dysplasia, autoimmune vasculitis, and neurofibromatosis [4].

The presence of collateral blood supply dictates the onset of clinical symptoms. The presenting symptoms are manifestations of interrupted blood supply to the upper extremity. Generally, symptoms include muscle fatigue, arm claudication, pain at rest, and finger necrosis [1]. Acute occlusion usually presents with a painful, cold, and pulseless limb [3]. Subclavian artery thrombosis originating proximally to the origin of the vertebral artery manifests as vertebrobasilar insufficiency. Neurological signs of vertebrobasilar insufficiency include diplopia, syncope, ataxia, drop attacks, vertigo, dysarthria, dizziness, nystagmus, tinnitus, and facial sensory deficits. A cardiovascular examination may reveal unequal blood pressure in both arms [1]. The interarm systolic blood pressure difference of <10 mmHg indicates a 99% negative predictive value [5]. However, an increase in the systolic cut off to 15-20 mmHg will raise the positive predictive value by 67%-100%, respectively. It will also decrease the negative predictive value by 100% [6,7]. The findings on pulse examination include absent or diminished axillary, radial, and brachial pulses. Other clinical findings such as gangrenous skin changes of the fingers and splinter hemorrhages of the nail beds are seen in late-diagnosed cases [1]. The most common complications of subclavian artery thrombosis include gangrenous limb and basilar stroke [1]. Various prodromes and manifestation may be evident in atherosclerotic occlusions [3].

Imaging remains the gold standard for diagnosis [2]. However, the initial workup of patients should include a complete blood count, prothrombin time, antithrombin III levels, alpha-macroglobulin levels, plasminogen levels, clotting factors, protein C/S, Factor V Leiden, and factor II C20210-a levels [3]. Lab investigations are usually of unremarkable yield. Duplex ultrasound with the color flow is the choice of investigation for noninvasive imaging. Computed tomography angiography and magnetic resonance angiography are required when an intervention is necessary [1]. In particular, multidetector computed tomography is useful to detect thoracic outlet abnormalities. A venous runoff should also be investigated due to a risk of co-existing subclavian vein thrombosis. An echocardiogram should also be conducted to rule out sources of arterial thrombi [3].

Therapeutic intervention is only indicated in symptomatic patients. The nonoperative treatment options provide limited benefit with limb-threatening ischemia. Medical therapeutic options include aspirin, clopidogrel, 3-hydroxy-3-methylglutaryl coenzyme A reductase inhibitors, angiotensin-converting enzyme inhibitors, and angiotensin receptor blockers [1]. They are used to supplement percutaneous or surgical treatment. Surgical options to correct pathology include axillary-axillary bypass, carotid-subclavian bypass, and transposition of the subclavian artery [1]. However, percutaneous transluminal angioplasty with stenting is currently the best modality for relieving the thrombus [8]. Contraindications for surgical intervention include inadequate distal runoff, inadequate vessel size, enhanced collateral circulation of the occluded area, and medical health contraindications [3].

Our patient presented with a gangrenous right distal extremity. This presentation is a complication of late-diagnosed subclavian artery thrombosis. As opposed to literature findings, our patient had disease involvement of the right subclavian artery. He also did not present with symptoms of vertebrobasilar insufficiency. This was due to the distal occlusion of the right subclavian artery after giving off the vertebral and internal thoracic branches. Co-existing risk factors such as diabetes and smoking were also present in our patient. We believe the chronic history of smoking, unmanaged diabetes, and unregulated diet predisposed our patient to right subclavian thrombus formation. Our case makes it quite evident that factors such as smoking, diabetes, hypertension, and hyperlipidemia predispose an individual to thrombosis. Therefore, we recommend that these risk factors must be managed optimally. Furthermore, the patient must be educated and counseled extensively regarding the consequences of continued smoking on the progression of atherosclerosis. In diabetics, strict glycemic control must be advised with HbA1c < 7%.

## Conclusions

Subclavian artery thrombosis seldom presents symptomatically and remains underdiagnosed by physicians. Our case presented with right distal extremity gangrene as a late-onset complication. This presentation emphasizes the importance of timely diagnosis and intervention. Co-existing multiple risk factors such as smoking and untreated diabetes are known to accelerate the underlying thrombogenic pathogenesis. In addition to the requirements of early medical and surgical interventions, our case also highlights the importance of optimally managing the underlying risk factors.
